# Multimodal analgesic regimen is associated with earlier bowel recovery in infants undergoing liver transplantation: a cohort study

**DOI:** 10.3389/fmed.2026.1962570

**Published:** 2026-10-23

**Authors:** Ling Zhu, Jiabei He, Leiqing Gao, Yuxiao Deng, Bo Qi

**Affiliations:** 1 Department of Anesthesiology, Renji Hospital, Shanghai Jiao Tong University School of Medicine, Shanghai, China; 2 Key Laboratory of Anesthesiology, Ministry of Education, Shanghai Jiao Tong University, Shanghai, China; 3 Department of Liver Surgery, Renji Hospital, Shanghai Jiao Tong University School of Medicine, Shanghai, China; 4 Department of Critical Care Medicine, Renji Hospital, Shanghai Jiao Tong University School of Medicine, Shanghai, China

**Keywords:** bowel recovery, infant, multimodal analgesia, postoperative pain, transplant surgery

## Abstract

**Purpose:**

This study aims to compare the effects of a multimodal analgesia regimen including sufentanil, esketamine, and dexmedetomidine with the traditional morphine-only analgesia approach on postoperative bowel recovery in pediatric liver transplant patients.

**Methods:**

This is a single-center retrospective cohort study. The medical records of a total of 82 infants who underwent liver transplantation at Renji Hospital, from September 2022 to March 2023 were included. Patients were divided into two groups based on the postoperative analgesia regimen: the morphine group (M group), which used morphine only, and the multimodal analgesia group (MMA group), which used sufentanil in combination with esketamine and dexmedetomidine.

**Results:**

The patients’ ages ranged from 4 to 12 months, and the mean preoperative pediatric end-stage liver disease (PELD) scores were 18.7 and 21.9. General linear model analysis revealed that the MMA group had a significantly shorter time to first bowel movement and shorter mechanical ventilation duration. Multivariate analysis further indicated that the use of multimodal analgesia significantly accelerated time to first defecation by approximately 20.6 h.

**Conclusion:**

Compared to using morphine alone for postoperative analgesia, the combination of sufentanil, esketamine, and dexmedetomidine for postoperative analgesia in liver transplant infants is more beneficial for brecovery and facilitates early postoperative extubation.

## Introduction

Liver transplantation is the most effective treatment for end-stage liver disease in both pediatric and adult patients (1). However, preoperative malnutrition, hypoalbuminemia, ascites, operative procedure, ischemia-reperfusion injury, anesthesia and postoperative managements make gastrointestinal dysfunction a common complication after transplantation (2). Notably, the postoperative gastrointestinal dysfunction (POGD) prolongs hospital stay, increases medical costs, and lowers the 1-year survival rate of patients (3, 4). Therefore, promoting gastrointestinal function recovery is a significant challenge for liver transplant patients.

One major factor contributing to POGD is postoperative pain management (5, 6), especially in pediatric patients. On the one hand, they cannot adequately express pain sensations which results in difficulties in pain assessment (7, 8). On the other hand, concerns about medication side effects in pediatric treatment have led to postoperative pain management techniques in children being relatively less mature than that in adults (9). Currently, guidelines for postoperative pain management in pediatric liver transplantation primarily recommend opioid-based regimens (10). However, opioids, by binding to gastrointestinal *μ*-opioid receptors, can affect gastrointestinal sensory and motor functions, increase non-propulsive contractions, reduce gastrointestinal secretion, inhibit intestinal transport, and increase fluid absorption (11). Together, these effects can suppress normal gastrointestinal activity and disrupt its recovery (7).

Multimodal analgesia refers to the use of multiple medications, physical therapies, and cognitive strategies to treat pain (12). It has become a recommended strategy for managing acute pain in hospitalized children, as it reduces opioid use and may contribute to enhanced recovery. However, studies evaluating multimodal analgesia protocols in pediatric liver transplantation are limited. This retrospective study aims to analyze clinical data from our center to compare the effects of a multimodal analgesia regimen including sufentanil, esketamine, and dexmedetomidine with the traditional morphine-only analgesia approach on postoperative bowel recovery in pediatric liver transplant recipients. The goal is to accelerate perioperative recovery, reduce complications, and improve long-term outcomes in pediatric liver transplantation.

## Methods

### Ethics statement

All procedures in this research were conducted in accordance with the Declaration of Helsinki and relevant clinical research regulations and guidelines in China. Ethical approval for the study was obtained from the Biomedical Ethics Committee of Renji Hospital, Shanghai, China (LY2024-315-C). The informed consent was waived by the Ethics Committee as only de-identified information was derived from the electronic medical records. This study is registered with Chinese Clinical Trial Registry Registration: https://www.chictr.org.cn/ (ChiCTR2500099054).

### Study design

This is a single-center retrospective before-and-after cohort study that included 82 children (≤ 2 yrs) who underwent living donor liver transplantation (LDLT) at Renji Hospital, from September 2022 to March 2023. Patients treated before November 10, 2022 received the morphine-based protocol, whereas those treated on or after November 10, 2022 received the newly implemented multimodal analgesic protocol. The exclusion criteria included: 1) Children who underwent reoperation within 48 h after liver transplantation (reoperation reasons include bleeding, thrombosis, bile leakage, bile duct stricture, infection, acute rejection, graft failure, etc.); 2) Children who were not transferred to the ICU after surgery; 3) Children who died within 3 days of ICU admission.

### Perioperative management

All children received transplantation underwent general anesthesia. All pediatric patients were fasted according to standard pediatric fasting guideline, which is 2 h for clear liquids, 4 h for breast milk, 6 h for non-human milk/ light meal, and 8 h for fatty meal. No bowel preparation, laxatives, or enemas were used preoperatively. After being admitted to the operating room, peripheral oxygen saturation (SpO_2_) and electrocardiography monitoring were applied. Then, 6–8 vol.% sevoflurane with 100% oxygen inhalation induction was conducted. When the recipient lost consciousness, a peripheral venous catherter was placed. Through it, intravenous anesthetic agents, including midazolam, and sufentanil were administered. Intubation was facilitated with rocuronium using a video laryngoscope.

Auscultating the bilateral lungs was important to locate the endotracheal tube. All patients received pressure-controlled ventilation after intubation. Mechanical ventilation was initiated with a tidal volume of 8 mL/kg, and the ventilatory frequency was controlled to maintain an end-tidal pressure of carbon dioxide up to 35–40 mmHg. Positive end expiration pressure (PEEP) was set at the level of 5 cmH_2_O.

Ultrasound-guided arterial puncture and right central vein puncture were performed to constantly monitor arterial pressure and central venous pressure (CVP). Pulse index Continuous Cardiac Output (PICCO) catheter was placed in the femoral artery to provide complete hemodynamic monitoring. An insulation blanket was laid under the patient to keep the body temperature above 36 °C. Anesthesia was maintained using sevoflurane, rocuronium, and sufentanil. Other monitoring procedures included end-expiratory carbon dioxide, body temperature, and urine volume. Blood gas was routinely analyzed at different phases of the surgery, including the preoperative, anhepatic, and new hepatic phase and the end of operation. Balanced crystalloids were used as the primary maintenance fluid. Albumin was administered for intravascular volume expansion when clinically indicated, based on an integrated assessment of blood pressure, central venous pressure, PICCO-derived hemodynamic parameters, urine output, ongoing blood loss, and blood gas results. Blood transfusion was given when hemoglobin was below 7 g/dL. Vasoconstrictor drugs were used intraoperatively, including ephedrine, epinephrine, and norepinephrine, to maintain blood pressure. The patients were sent to the intensive care unit (ICU) after surgery.

Before Nov 10th, 2022, postoperative analgesia was routinely provided using continuous morphine infusion. Patients received continuous morphine infusion via an intravenous pump (0.06 mg/kg/h). Eligible patients treated during this period constituted the morphine group (M group). On and after Nov 10th, 2022, multimodal analgesia (MMA) regimen was implemented. Patients in MMA group received analgesics via an infusion pump using a regimen of sufentanil (1 μg/kg) + esketamine (1 mg/kg) + dexmedetomidine (2 μg/kg) + dolasetron (2 mg/kg) diluted to 100 mL. Background dose: 1.5 mL/h; bolus dose: 1.5 mL; lockout time: 15 min. Both groups started postoperative analgesia on admission to the ICU and continued until 48 h after surgery. Postoperative care in the ICU was provided according to standard hospital protocols, which included specific guidelines for postoperative feeding protocols, use of prokinetic agents, sedation strategies, and ventilator weaning practices. Enteral feeding was typically initiated within 12–24 h after surgery based on the patient's hemodynamic stability and absence of contraindications. Prokinetic agents (e.g., metoclopramide or erythromycin) were administered at the discretion of the attending physician, primarily in cases of suspected gastroparesis or delayed gastric emptying. Postoperative fluid management was weight-adjusted and guided by hemodynamic status, urine output enteral intake, drainage losses, and blood gas analysis. Sedation depth was evaluated using the State Behavioral Scale (SBS). Propofol was infused at 1–3 mg/kg/h to achieve the institutional target of SBS < −1; if sedation remained inadequate at 3 mg/kg/h, midazolam was added at 0.2 mg/kg/h. Ventilator weaning followed a standardized protocol that included assessment of extubation readiness based on clinical parameters such as adequate oxygenation, hemodynamic stability, and acceptable respiratory mechanics.

### Outcome measures and data collection

The primary outcome is the time to first defecation after surgery, based on nuring records in medical chart. The secondary outcomes included postoperative mechanical ventilation time, ICU stay, time to remove the endotracheal tube, gastric tube, abdominal drainage tube, and urinary catheter, changes in postoperative liver enzyme levels, all obtained from routine medical records in the electronic medical chart. Other parameters including baseline characteristics, anesthesia and surgery related information and postoperative data were also obtained.

### Statistical analysis

Data were analyzed using IBM SPSS Statistics version 25.0. Continuous variables with a normal distribution were analyzed using the Student's t-test and presented as mean (M) ± standard deviation (Std). Non-normally distributed variables were analyzed using the Mann–Whitney U test and expressed as median [interquartile range (IQR)]. Categorical variables were compared using the chi-square test or Fisher's exact test, with results reported as *n* (%). Variables with statistical significance in preoperative and intraoperative data were further analyzed for their correlations with primary and secondary outcomes. Pearson correlation was applied for linear relationships (correlation coefficients denoted as r), and Spearman correlation for non-linear relationships (correlation coefficients denoted as r’). Scatter plots were used to visualize the relationships. Outcome measures were analyzed using generalized linear models (GLM), with results presented as estimate marginal means (EMM) ± Std. Repeated measures at different postoperative time points were compared using generalized estimating equations (GEE), with outcomes also expressed as EMM ± Std. A *P*-value < 0.05 was considered statistically significant.

## Results

### Demographics

82 infants who underwent liver transplantation at Renji Hospital, Shanghai, China, from September 2022 to March 2023 were included, of which 41 received morphine analgesia, and 41 received multimodal analgesia. The mean age was 6.7 months. About half of them were male. The average weight was 6.9 kg in M group and 7.3 in MMA group, and the average height was 65.3 cm. No statistically significant differences were found between the two groups in age, sex, height, or weight (Table 1). The mean preoperative pediatric end-stage liver disease (PELD) scores were 18.7 in the M group and 21.9 in the MMA group, and the difference was not statistically significant. Among the patients, 6 had PELD scores below 10, and 24 had scores below 16. There were no statistically significant differences between the two groups in terms of preoperative PELD scores or the proportions of children with lower PELD scores (Table 1).

**Table 1:** Demographics.

Characteristics	M Group *N* = 41	MMA Group *N* = 41	*P* Value
Age, month(s), M ± Std	6.6 ± 1.9	6.8 ± 2.0	0.693
Male, *n* (%)	22 (53.7)	23 (56.1)	0.824
Weight, kg, M ± Std	6.9 ± 1.3	7.3 ± 1.3	0.157
Height, cm, M ± Std	65.3 ± 4.5	65.3 ± 3.2	0.932
PELD score, M ± Std	18.7 ± 9.3	21.9 ± 10.4	0.153
PELD score, <10, *n* (%)	5 (12.2)	1 (2.4)	0.203
PELD score, <16, *n* (%)	13 (31.7)	11 (26.8)	0.627

PELD score = Pediatric End-Stage Liver Disease score, M = Mean, Std = Standard deviation, Min = Minimum, Max = Maximum, N = number.

### Perioperative information

No statistically significant differences were observed between the two groups in surgery time (*P* = 0.473), anesthesia time (*P* = 0.977), anhepatic time (*P* = 0.056), blood transfusion volume (*P* = 0.954), urine output volume (*P* = 0.321), or intraoperative sufentanil dosage (*P* = 0.391) (Table 2). However, the MMA group received significantly more albumin intraoperatively (21.2 ± 5.6 g) compared to the M group (16.2 ± 6.7 g, *P* = 0.001, Table 2).

**Table 2:** Perioperative data.

Parameters	M Group *N* = 41	MMA Group *N* = 41	*P* Value
Surgery time, min, M ± Std	348.1 ± 64.2	339.3 ± 43.1	0.473
Anesthesia time, min, M ± Std	414.3 ± 69.0	414.7 ± 42.2	0.977
Anhepatic phase, min, M ± Std	40.4 ± 8.4	37.3 ± 5.9	0.056
Blood transfusion, U, Med (IQR)	1.0 (0.0)	1.0 (0.0)	0.954
Albumin infusion, g, M ± Std	16.2 ± 6.7	21.2 ± 5.6	0.001*
Urine output, mL, Med (IQR)	160.0 (100.0)	180.0 (125.0)	0.321
Sufentanil use, ug, M ± Std	70.7 ± 14.4	68.1 ± 13.2	0.391

M = Mean, Std = Standard deviation, Min = Minimum, Max = Maximum, Med = Median, N = number, % = percent.

* *P* < 0.01.

### Postoperative gastrointestinal function recovery

All children were transferred to the ICU with endotracheal tubes in place for further treatment. The generalized linear model for comparison shows that the time to first defecation was significantly shorter in the MMA group (27.8 ± 2.3 h) compared to the M group (48.6 ± 2.9 h), with a statistically significant difference (mean difference and 95% CI: 20. 778 [14.381, 27.174], *P* < 0.001, Table 3). The MMA group was also associated with significantly shorter mechanical ventilation time (20.8 ± 3.1 vs. 34.5 ± 3.6, mean difference and 95% CI: 13.612 (2.092, 25.131), *p* = 0.021). Gastric and abdominal drainage tube placement duration were comparable between the two groups (Figure 1). Even though the MMA group (109.7 ± 2.9) was associated with shorter ICU stay than M group (120.1 ± 3.4), the difference was statistically insignificant (*P* = 0.054). No between-group difference was found in urinary catheter time (*P* = 0.686, Table 3).

**Table 3:** Outcome measures.

Parameters	M Group *N* = 41	MMA Group *N* = 41	Mean Difference(95% CI)	*P* Value	
Time to first defecation, h, M ± Std	48.6 ± 2.9	27.8 ± 2.3	20.778 (14.381, 27.174)		<0.001**
Gastric tube placement, d, M ± Std	4.1 ± 0.1	4.1 ± 0.1	0.063 (−0.316, 0.441)		0.746
Abdominal drainage tube placement, d, M ± Std	5.5 ± 0.3	5.0 ± 0.3	0.547 (−0.182, 1.276)		0.141
Mechanical ventilation duration, h, M ± Std	34.5 ± 3.6	20.8 ± 3.1	13.612 (2.092, 25.131)		0.021*
ICU stay, h, M ± Std	120.1 ± 3.4	109.7 ± 2.9	10.476 (−0.184, 21.136)		0.054
Urinary catheter time, h, M ± Std	103.9 ± 3.8	101.4 ± 3.2	5.478 (−4.991, 15.947)		0.686

M = Mean, Std = Standard deviation, Min = Minimum, Max = Maximum.

* *P* < 0.05.

** *P* < 0.001.

**Figure 1:**
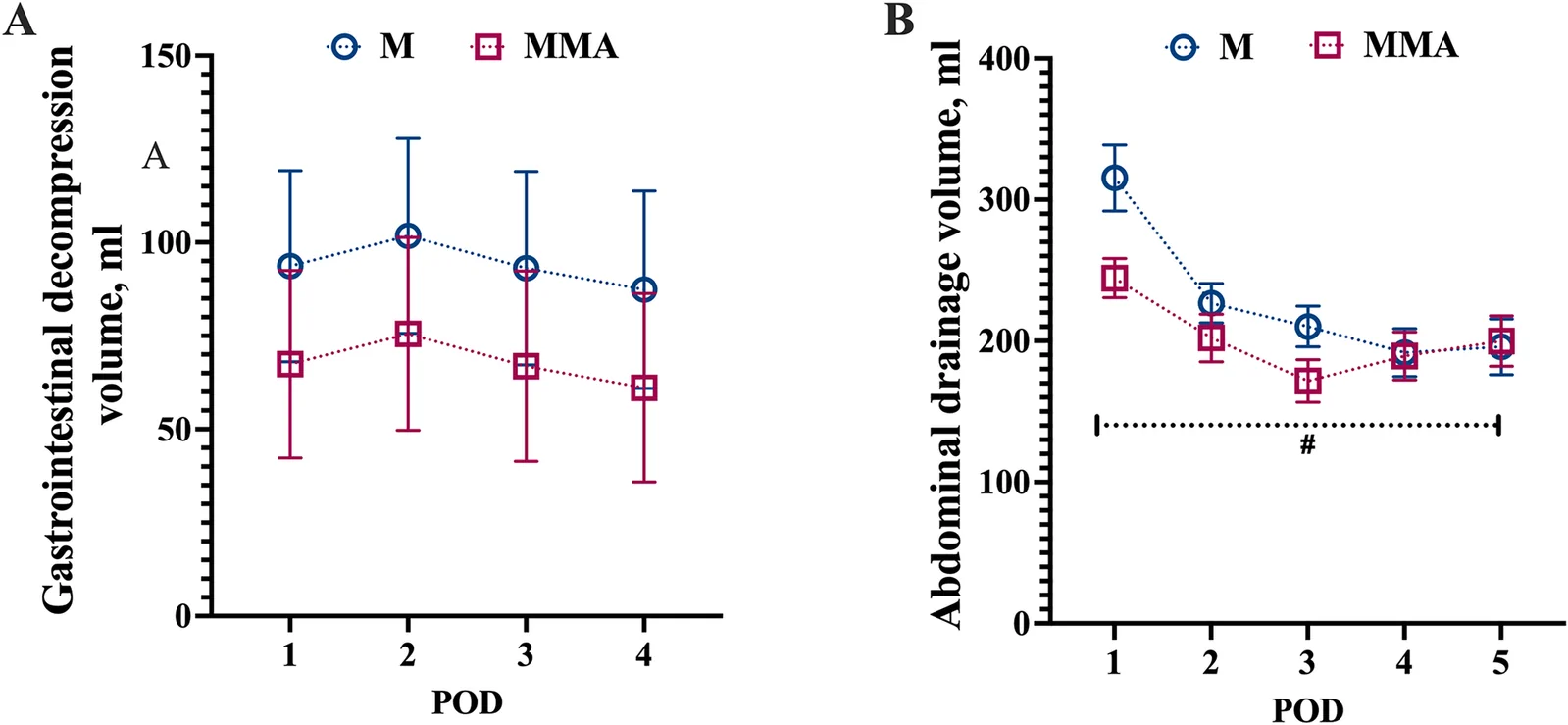
Gastrointestinal decompression volume and abdominal drainage volume. Inter-group comparison of morphine and multimodal analgesia group between gastrointestinal decompression volume **(A)** and abdominal drainage volume **(B)** within first 4 or 5 postoperative days.

In addition, no significant differences were found between the M and MMA groups for laboratory results, including hemoglobin, white blood cells, platelets, total bilirubin, ALT, AST, albumin, creatinine levels and INR on postoperative day 3 (Figure 2).

**Figure 2:**
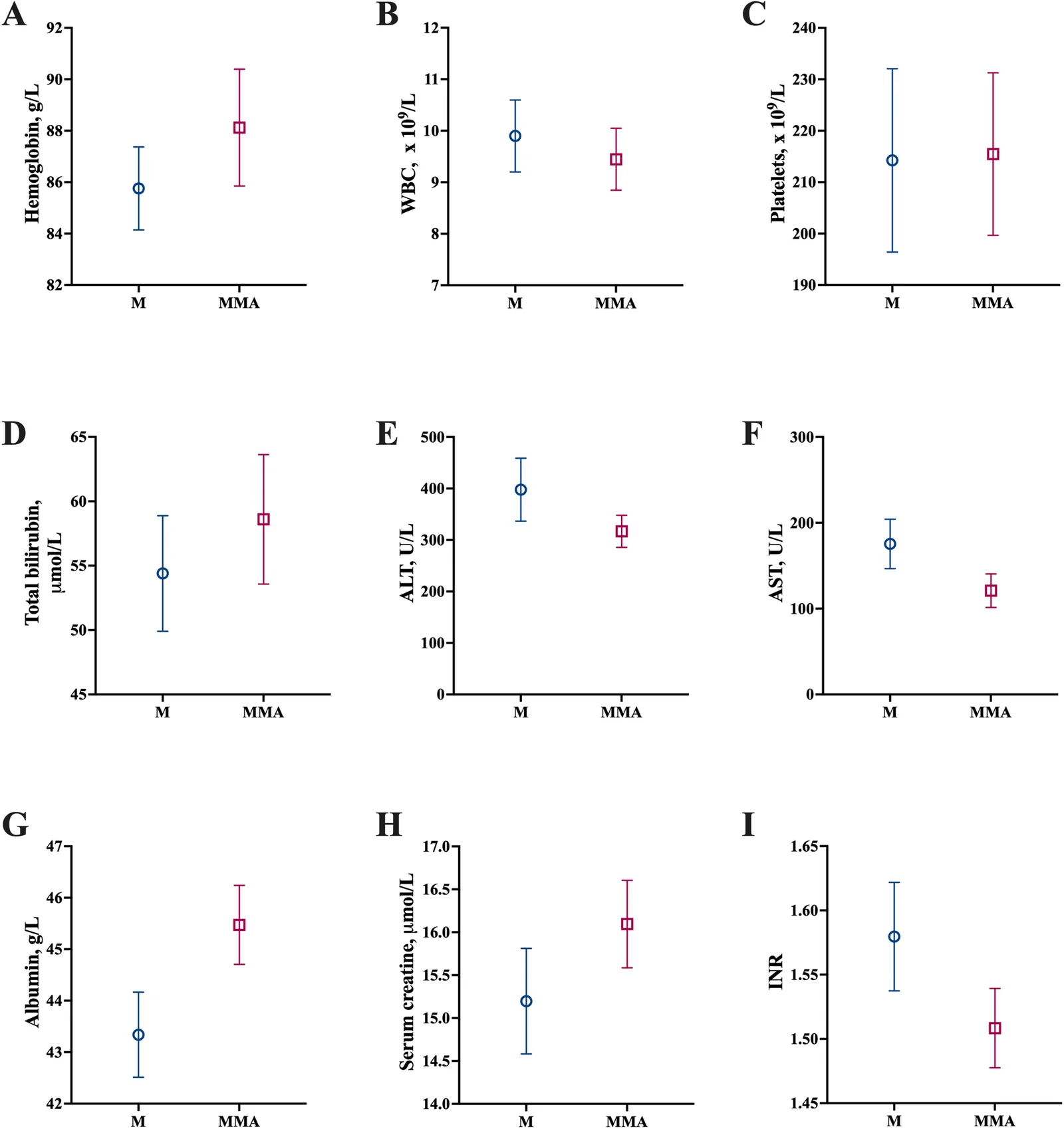
Postoperative laboratory results on day 3. Inter-group comparison of hemoglobin **(A)**, white blood cells **(B)**, platelets **(C)**, total bilirubin **(D)**, ALT **(E)**, AST **(F)**, albumin **(G)**, creatinine levels **(H)** and INR **(I)** on postoperative day 3. WBC = white blood cells, ALT = alanine transaminase, AST = aspartate aminotransferase, INR = international normalized ratio.

### Multivariate analysis

A multivariate analysis was conducted to determine potential factors affecting the recovery of bowel function (measured as time to first defecation) in pediatric liver transplant recipients (Table 4). The analysis included preoperative, intraoperative, and postoperative parameters. Preoperative parameters included sex, age (in months), height, weight, and PELD score. Intraoperative parameters included blood transfusion volume, albumin administration, urine output volume, anhepatic time, anesthesia time, and intraoperative sufentanil dosage. Postoperative parameters included mode of analgesia (MMA vs. M). This statistical model (adjusted R^2^ is 0.413, Durbin-Watson value is 2.116, F-value is 5.753, *P* < 0.001) showed that multimodal analgesia significantly shortened the time (by 20.555 h) to first defecation compared to morphine-only analgesia (95% CI: −26.512 to −14.597, * * *P* < 0.001).

**Table 4:** Multivariate analysis.

Parameters	Unstandardized Coefficients	Std. Error	Standardized Coefficients	t	*P* Value	95% CI	
Sex	0.644	2.817	0.022	0.229	0.820	−4.976	6.264
Age, month(s)	0.26	0.954	0.034	0.272	0.786	−1.644	2.163
Weight, kg	−0.361	2.052	−0.032	−0.176	0.861	−4.456	3.733
Height, cm	0.138	0.744	0.036	0.185	0.854	−1.347	1.623
PELD score	0.255	0.142	0.171	1.801	0.076	−0.027	0.537
Blood transfusion, U	0.028	2.947	0.001	0.009	0.993	−5.852	5.907
Albumin, g	0.222	0.224	0.099	0.989	0.326	−0.225	0.669
Urine output, mL	−0.016	0.013	−0.112	−1.198	0.235	−0.042	0.011
Anhepatic phase, min	−0.221	0.201	−0.11	−1.104	0.274	−0.622	0.179
Anesthesia time,min	−0.046	0.029	−0.178	−1.572	0.12	−0.105	0.012
Sufentanil use, ug	0.193	0.122	0.179	1.578	0.119	−0.051	0.437
MMA (*vs* M)	−20.555	2.986	−0.699	−6.883	<0.001*	−26.512	−14.597

PELD score = Pediatric End-Stage Liver Disease score. Adjusted R Square = 0.413, Durbin-Watson value = 2.116, F value = 5.753, *P* < 0.001.

## Discussion

Postoperative gastrointestinal dysfunction is a major and widely recognized complication, as it prolongs hospital stays, increases healthcare costs, and hampers survival rates (3, 4). In this retrospective study, we demonstrated that compared to morphine only regimen, multimodal postoperative analgesia consisting of sufentanil, ketamine and dexmedetomidine ia associated with earlier recovery of gastrointestinal function and earlier tracheal extubation.

The PELD scoring system is commonly used to evaluate the condition of children before transplantation, with higher scores indicating poorer prognosis (13). In this study, the mean PELD score was above 18, reflecting advanced disease severity in the study population. We further compared the proportion of children with PELD scores below 10 and below 16 between the two groups and found no significant differences, suggesting comparable levels of pretransplant severity.

Optimizing postoperative pain management in pediatric liver recipients is widely recognized as an important factor in promoting gastrointestinal function recovery, and improving quality of life (14, 15). Multimodal analgesia, a key component of enhanced recovery after surgery (ERAS), has been shown to improve pain control and gastrointestinal function in numerous studies (16). However, due to the complexity of liver transplantation and the difficulty in assessing pain in pediatric patients, studies examining multimodal analgesia in pediatric liver transplantation remain limited (6, 8). Esketamine provides effective sedative and analgesic effects and is widely used in pediatric anesthesia due to its mild hemodynamic stimulation and minimal respiratory depression (17). Dexmedetomidine, a sedative with adjuvant anesthetic properties, enhances analgesic effects when combined with other analgesics and can reduce serum levels of inflammatory cytokines and stress-related molecules (18). Therefore, this study investigated the use of a regimen combining sufentanil, esketamine, and dexmedetomidine for postoperative analgesia in pediatric liver transplant patients.

Compared to opioids-only analgesia, MMA regimen significantly shortened the time to first defecation, by approximately 20.6 h. This accelerated recovery of gastrointestinal function may be partly attributed to the reduced use of opioids, as opioids are known to delay gastrointestinal motility (19). Patients in the M group received a mean cumulative morphine dose of approximately 19.9 mg over the first 48 postoperative hours, whereas those in the MMA group received an average of 5.3 µg of sufentanil. Based on standard opioid conversion ratios (sufentanil 1 µg ≈ 1 mg of intravenous morphine), this dose is equivalent to approximately 5.3 mg of morphine milligram equivalents (MME). In other words, the multimodal analgesia regimen was associated with an approximately 74% reduction in postoperative opioid exposure.

Another contributing factor may be the inclusion of dexmedetomidine and esketamine in the regimen. Our findings are consistent with previous evidence suggesting that both agents can facilitate postoperative gastrointestinal recovery (20, 21). A large-scale multicenter randomized controlled trial in elderly patients undergoing abdominal surgery reported that the use of dexmedetomidine during surgery significantly reduced the time to first bowel movement and first flatus (22). A meta-analysis of 22 randomized controlled trials involving 2,566 patients confirmed that dexmedetomidine significantly improved gastrointestinal recovery in patients undergoing non-cardiac surgery (23). Similar benefits have also been reported for esketamine (24). For example, a study of patients undergoing total laparoscopic hysterectomy showed that an opioid-sparing anesthesic strategy based on esketamine resulted in lower visual analog scale (VAS) scores and a lower incidence of nausea and vomiting within the first 24 h after surgery compared to opioid-based anesthesia (25). However, it should be noted that dexmedetomidine and esketamine were administered at relatively low doses in our MMA protocol, therefore, their individual effects cannot be determined from the present study.

Compared to morphine analgesia, the combination of sufentanil, esketamine, and dexmedetomidine also significantly reduced the mechanical ventilation time with a tracheal tube by approximately 13.6 h. Opioids can suppress coughing and respiratory drive and hinder bronchial mucus secretion (26). Therefore, the combined use of sufentanil, esketamine, and dexmedetomidine in this study may have contributed to early extubation by reducing opioid consumption. Indeed, a review of rapid cardiac care for adult heart surgery patients indicated that lower opioids dose helps reduce extubation time and shorten postoperative ICU stay (27). Moreover, dexmedetomidine has been widely used to facilitate extubation in clinical practice, because it provides adequate sedation without causing significant respiratory depression (28, 29). In a randomized, double-blind, placebo-controlled, cross-over study, K Jonkman et al. found that esketamine effectively countered remifentanil-induced respiratory depression by increasing remifentanil-reduced ventilatory CO_2_ chemosensitivity (30). These pharmacological properties may have contributed to earlier extubation in pediatric liver transplant patients. However, this finding should be interpreted with caution. Given the retrospective nature of the study, the individual contributions of reduced opioid exposure, dexmedetomidine and esketamine, and other aspects of postoperative sedation management cannot be distinguished. Although postoperative sedation management was conducted according to a standardized ICU protocol, detailed data on postoperative sedatives, pain and sedation assessments were not consistently available. Consequently, potential influence of postoperative sedation management as a confounding factor cannot be excluded. Therefore, the observed reductions in time to first defecation and mechanical ventilation duration should be interpreted as associations with the overall multimodal analgesic regimen rather than effects attributable to individual component.

MMA group received higher albumin transfusion than M group during surgery. The difference is partly attributed to a slightly higher weight in the MMA group (Table 1), as intraoperative albumin transfusion is calculated based on body weight. Serum albumin is an important biochemical marker of nutritional status, and higher perioperative serum albumin levels are associated with better recovery, fewer complications, and improved survival after major surgery (31–34). However, in the present study, laboratory findings, including liver function indicators on postoperative day 3 (Figure 2), showed comparable liver function between the two groups. Moreover, albumin transfusion was not significantly correlated with ICU stay or the durations of gastric tube and abdominal drain retention, and transfusion variables were included in the multivariable analysis, suggesting a minimal impact of the differences in albumin transfusion.

Several limitations exist in this study. First, the retrospective before-and-after design may be susceptible to temporal bias. Even though postoperative management, including sedation criteria, extubation criteria, feeding strategies, and bowel management were carried out in accordance with standard hospital protocols, unmeasured temporal changes in clinical practice cannot be completely excluded. Second, several potentially relevant perioperative and postoperative variables, including intraoperative hemodynamic parameters, use of prokinetic agents, pain and sedation scores, and postoperative sedative doses, were not consistently available for analysis. These limitations may have introduced residual confounding and should be considered when interpreting the observed between-group differences. Third, a small sample size of this study limits the ability to detect significant associations for several secondary outcomes. The quantitative relationship between albumin consumption and recovery profiles, like ICU stay, gastric tube retention time, and abdominal drainage tube retention time cannot be well established. Fourth, time to first defecation was used in this study as a main indicator, but it alone may not fully reflect the overall recovery of gastrointestinal function. Lack of other parameters, such as time to first flatus, tolerance of enteral feeding may restrict the comprehensiveness of our findings. Finally, this study mainly focused on early postoperative period and did not include long-term follow-up.

## Conclusion

A multimodal analgesia regimen based on sufentanil, esketamine, and dexmedetomidine was associated with faster recovery of gastrointestinal functions, and a shorter duration of mechanical ventilation in infants undergoing liver transplant. These findings underscore the clinical benefit of using multimodal analgesia for postoperative pain management in pediatric liver transplant patients.

## Data Availability

The raw data supporting the conclusions of this article will be made available by the authors, without undue reservation.

## References

[1] Bodzin AS Baker TB. Liver transplantation today: where we are now and where we are going. Liver Transpl. (2018) 24:1470–5. 10.1002/lt.25320 30080954

[2] Brubaker AL Sellers MT Abt PL Croome KP Merani S Wall A. US liver transplant outcomes after normothermic regional perfusion vs standard super rapid recovery. JAMA Surg. (2024) 159:677–85. 10.1001/jamasurg.2024.0520 38568597 PMC10993160

[3] Hibi T Wei Chieh AK Chi-Yan Chan A Bhangui P. Current status of liver transplantation in Asia. Int J Surg. (2020) 82(82S):4–8. 10.1016/j.ijsu.2020.05.071 32535264

[4] Kassel CA Wilke TJ Fremming BA Brown BA. 2021 clinical update in liver transplantation. J Cardiothorac Vasc Anesth. (2022) 36:4183–91. 10.1053/j.jvca.2022.05.027 35902314

[5] Arena S Di Fabrizio D Impellizzeri P Gandullia P Mattioli G Romeo C. Enhanced recovery after gastrointestinal surgery (ERAS) in pediatric patients: a systematic review and meta-analysis. J Gastrointest Surg. (2021) 25:2976–88. 10.1007/s11605-021-05053-7 34244952

[6] Smith HAB Besunder JB Betters KA Johnson PN Srinivasan V Stormorken A. 2022 society of critical care medicine clinical practice guidelines on prevention and management of pain, agitation, neuromuscular blockade, and delirium in critically ill pediatric patients with consideration of the ICU environment and early mobility. Pediatr Crit Care Med. (2022) 23:e74–e110. 10.1097/PCC.0000000000002873 35119438

[7] Zielinski J Morawska-Kochman M Zatonski T. Pain assessment and management in children in the postoperative period: a review of the most commonly used postoperative pain assessment tools, new diagnostic methods and the latest guidelines for postoperative pain therapy in children. Adv Clin Exp Med. (2020) 29:365–74. 10.17219/acem/112600 32129952

[8] Daverio M Von Borell F Ramelet A-S Sperotto F Pokorna P Brenner S. The nurse science section of the European society of, and C. Neonatal intensive, pain and sedation management and monitoring in pediatric intensive care units across Europe: an ESPNIC survey. Crit Care. (2022) 26:88. 10.1186/s13054-022-03957-7 35361254 PMC8969245

[9] Frizzell KH Cavanaugh PK Herman MJ. Pediatric perioperative pain management. Orthop Clin North Am. (2017) 48:467–80. 10.1016/j.ocl.2017.06.007 28870306

[10] Kim TW. Caudal morphine for pain relief in pediatric liver transplantation: did it help? Anesth Analg. (2005) 100:603. 10.1213/01.ANE.0000145321.44735.3B 15673914

[11] Dalal RS. Limiting opioid use for patients who are hospitalized for inflammatory bowel disease exacerbations. Inflamm Bowel Dis. (2025) 31:610–1. 10.1093/ibd/izae214 39301671

[12] Cozowicz C Zhong H Poeran J Illescas A Liu J Poultsides LA. The impact of multimodal analgesia in coronary artery bypass graft surgery-a population-based analysis. J Thorac Cardiovasc Surg. (2025) 169:105–113.e5. 10.1016/j.jtcvs.2023.11.035 38042402

[13] Sanchez MC D'Agostino DE. Pediatric end-stage liver disease score in acute liver failure to assess poor prognosis. J Pediatr Gastroenterol Nutr. (2012) 54:193–6. 10.1097/MPG.0b013e3182349a04 21886007

[14] Boric K Dosenovic S Jelicic Kadic A Batinic M Cavar M Urlic M. Interventions for postoperative pain in children: an overview of systematic reviews. Paediatr Anaesth. (2017) 27:893–904. 10.1111/pan.13203 28707454

[15] Sharek PJ Wayman K Lin E Strichartz D Sentivany-Collins S Good J. Improved pain management in pediatric postoperative liver transplant patients using parental education and non-pharmacologic interventions. Pediatr Transplant. (2006) 10:172–7. 10.1111/j.1399-3046.2005.00438.x 16573603

[16] Tian Q Wang H Guo T Yao B Liu Y Zhu B. The efficacy and safety of enhanced recovery after surgery (ERAS) program in laparoscopic distal gastrectomy: a systematic review and meta-analysis of randomized controlled trials. Ann Med. (2024) 56:2306194. 10.1080/07853890.2024.2306194 38279689 PMC10823895

[17] Zhang Y Chen R Tang S Sun T Yu Y Shi R. Diurnal variation of postoperative delirium in elderly patients undergoing esketamine anesthesia for elective noncardiac surgery: a randomized clinical trial. Int J Surg. (2024) 110:5496–504. 10.1097/JS9.0000000000001642 39275772 PMC11392167

[18] Grewal A. Dexmedetomidine: new avenues. J Anaesthesiol Clin Pharmacol. (2011) 27:297–302. 10.4103/0970-9185.83670 21897496 PMC3161450

[19] Kaur S Henthorn P Hendrickse A. Pain management for liver transplant recipients. Curr Opin Organ Transplant. (2023) 28:397–403. 10.1097/MOT.0000000000001102 37728190

[20] Chen L He W Liu X Lv F Li Y. Application of opioid-free general anesthesia for gynecological laparoscopic surgery under ERAS protocol: a non-inferiority randomized controlled trial. BMC Anesthesiol. (2023) 23:34. 10.1186/s12871-023-01994-5 36707777 PMC9881250

[21] Kaye AD Chernobylsky DJ Thakur P Siddaiah H Kaye RJ Eng LK. Dexmedetomidine in enhanced recovery after surgery (ERAS) protocols for postoperative pain. Curr Pain Headache Rep. (2020) 24:21. 10.1007/s11916-020-00853-z 32240402 PMC7223065

[22] Lu Y Fang P-P Yu Y-Q Cheng X-Q Feng X-M Wong GTC. Effect of intraoperative dexmedetomidine on recovery of gastrointestinal function after abdominal surgery in older adults: a randomized clinical trial. JAMA Netw Open. (2021) 4:e2128886. 10.1001/jamanetworkopen.2021.28886 34648009 PMC8517746

[23] Lai YC Wang WT Hung KC Chen JY Wu JY Chang YJ. Impact of intravenous dexmedetomidine on postoperative gastrointestinal function recovery: an updated meta-analysis. Int J Surg. (2024) 110:1744–54. 10.1097/JS9.0000000000000988 38085848 PMC10942148

[24] Zhang T Yue Z Yu L Li S Xie Y Wei J. S-ketamine promotes postoperative recovery of gastrointestinal function and reduces postoperative pain in gynecological abdominal surgery patients: a randomized controlled trial. BMC Surg. (2023) 23:74. 10.1186/s12893-023-01973-0 36997940 PMC10061816

[25] Liu J Yin J Yin J Zhou M Chen L Dong X. Effect of esketamine-based opioid-sparing anesthesia strategy on postoperative pain and recovery quality in patients undergoing total laparoscopic hysterectomy: a randomized controlled trail. Heliyon. (2024) 10:e24941. 10.1016/j.heliyon.2024.e24941 38317936 PMC10839621

[26] Steffens C Sung M Bastian LA Edelman EJ Brackett A Gunderson CG. The association between prescribed opioid receipt and community-acquired pneumonia in adults: a systematic review and meta-analysis. J Gen Intern Med. (2020) 35:3315–22. 10.1007/s11606-020-06155-9 32885375 PMC7661588

[27] Wong WT Lai VK Chee YE Lee A. Fast-track cardiac care for adult cardiac surgical patients. Cochrane Database Syst Rev. (2016) 2016:CD003587. 10.1002/14651858.CD003587.pub3 PMC6457798 27616189

[28] Siobal MS Kallet RH Kivett VA Tang JF. Use of dexmedetomidine to facilitate extubation in surgical intensive-care-unit patients who failed previous weaning attempts following prolonged mechanical ventilation: a pilot study. Respir Care. (2006) 51:492–6. 10.4187/respcare.06510492 16638158

[29] Buckley MS Smithburger PL Wong A Fraser GL Reade MC Klein-Fedyshin M. Dexmedetomidine for facilitating mechanical ventilation extubation in difficult-to-wean ICU patients: systematic review and meta-analysis of clinical trials. J Intensive Care Med. (2021) 36:925–36. 10.1177/0885066620937673 32627672

[30] Jonkman K Van Rijnsoever E Olofsen E Aarts L Sarton E Van Velzen M. Esketamine counters opioid-induced respiratory depression. Br J Anaesth. (2018) 120:1117–27. 10.1016/j.bja.2018.02.021 29661389

[31] Aronen M Viikari L Langen H Kohonen I Wuorela M Vuorinen T. The long-term prognostic value of serum 25(OH)D, albumin, and LL-37 levels in acute respiratory diseases among older adults. BMC Geriatr. (2022) 22:146. 10.1186/s12877-022-02836-8 35189828 PMC8860370

[32] Ni T Wen Y Wang Y Jiang W Sheng H Chen E. Association between albumin or prealbumin levels at different stages and prognosis in severe acute pancreatitis: a 5-year retrospective study. Sci Rep. (2022) 12:16792. 10.1038/s41598-022-21278-1 36202939 PMC9537333

[33] Ono R Horibata K. Cross-sectional study investigating the relationship between pit recovery time and serum albumin levels in bilateral lower extremity pitting oedema. BMJ Open. (2024) 14:e079327. 10.1136/bmjopen-2023-079327 38238047 PMC10806623

[34] Yang SP Wang TJ Huang CC Chang SC Liang SY Yu CH. Influence of albumin and physical activity on postoperative recovery in patients with colorectal cancer: an observational study. Eur J Oncol Nurs. (2021) 54:102027. 10.1016/j.ejon.2021.102027 34509088

